# Enhancement of Implant Osseointegration by High-Frequency Low-Magnitude Loading

**DOI:** 10.1371/journal.pone.0040488

**Published:** 2012-07-10

**Authors:** Xiaolei Zhang, Antonia Torcasio, Katleen Vandamme, Toru Ogawa, G. Harry van Lenthe, Ignace Naert, Joke Duyck

**Affiliations:** 1 Department of Prosthetic Dentistry, BIOMAT Research Cluster, University of Leuven, Leuven, Belgium; 2 Department of Mechanical Engineering, Division of Biomechanics and Engineering Design, University of Leuven, Leuven, Belgium; 3 Division of Advanced Prosthetic Dentistry, Tohoku University Graduate School of Dentistry, Sendai, Japan; 4 Institute for Biomechanics, ETH Zurich, Zurich, Switzerland; University of Notre Dame, United States of America

## Abstract

**Background:**

Mechanical loading is known to play an important role in bone remodelling. This study aimed to evaluate the effect of high- and low-frequency axial loading, applied directly to the implant, on peri-implant bone healing and implant osseointegration.

**Methodology:**

Titanium implants were bilaterally installed in rat tibiae. For every animal, one implant was loaded (test) while the other one was not (control). The test implants were randomly divided into 8 groups according to 4 loading regimes and 2 experimental periods (1 and 4 weeks). The loaded implants were subject to an axial displacement. Within the high- (HF, 40 Hz) or low-frequency (LF, 8 Hz) loading category, the displacements varied 2-fold and were ranked as low- or high-magnitude (LM, HM), respectively. The strain rate amplitudes were kept constant between the two frequency groups. This resulted in the following 4 loading regimes: 1) HF-LM, 40 Hz-8 µm; 2) HF-HM, 40 Hz-16 µm; 3) LF-LM, 8 Hz-41 µm; 4) LF-HM, 8 Hz-82 µm. The tissue samples were processed for resin embedding and subjected to histological and histomorphometrical analyses. Data were analyzed statistically with the significance set at *p*<0.05.

**Principal Findings:**

After loading for 4 weeks, HF-LM loading (40 Hz-8 µm) induced more bone-to-implant contact (BIC) at the level of the cortex compared to its unloaded control. No significant effect of the four loading regimes on the peri-implant bone fraction (BF) was found in the 2 experimental periods.

**Conclusions:**

The stimulatory effect of immediate implant loading on bone-to-implant contact was only observed in case of high-frequency (40 Hz) low-magnitude (8 µm) loading. The applied load regimes failed to influence the peri-implant bone mass.

## Introduction

Bone tissue is metabolically active in adapting its mass, shape and structure to mechanical stimuli through remodelling. Mechanical loading has been proven to direct the differentiation of mesenchymal stem cells towards the osteoblastic lineage and has therefore been introduced to facilitate fracture healing and to improve bone quality [1], [2], [3], [4]. In animal studies, the anabolic effect of mechanical loading on bone tissue has been evidenced when applied at both high- and low-frequency [2], [5].

The effect of mechanical loading on bone regeneration and adaptation also applies to bone around biomaterials, and more specifically around titanium implants [6], [7], [8]. Findings from *in vivo* studies have shown that force- [9], [10], [11] or displacement- [7], [8], [12], [13] controlled mechanical loading at low-frequency (<10 Hz), when applied directly onto an implant, can improve bone formation in the peri-implant region and can therefore contribute to implant osseointegration. Recent research also revealed that high-frequency loading (>10 Hz), applied via whole body vibration, can lead to increased bone formation in the peri-implant surroundings and ultimately to an improved osseointegration [14], [15], [16]. An early investigation revealed a pronounced peri-implant bone response to both high- (20 Hz) and low-frequency (1 Hz) loading [17].

Despite the above notions, further research on the peri-implant tissue response to mechanical loading at high- *versus* low-frequency is warranted due to the variety of animal models and of loading modes (*i.e.* the loading was directly or indirectly applied onto the implant) used in the aforementioned studies [5]–[11]. Furthermore, the impact of the loading magnitude in high- *versus* low-frequency loading regimes is only partly unraveled. There is evidence that up to a certain limit, the load-induced bone gain is determined by the loading magnitude in a low-frequency regime [18], [19], [20], [21]. In case of high-frequency stimulation, however, the loading magnitude is reported to be less relevant [5], [22]. To explore the therapeutic potential of high-frequency mechanical loading in titanium implant healing, it is valuable to fill this knowledge gap and hence to determine appropriate loading strategies.

By use of a rat tibia model and a displacement-controlled loading device, the present study aimed to investigate the influence of controlled mechanical loading, directly applied to the implant and immediately after implant installation, at high- *versus* low-frequency on peri-implant bone (re)modelling and implant osseointegration. It was hypothesized that (i) the peri-implant bone responds to high- and low-frequency loading; and that (ii) this bone response depends on the applied loading magnitude.

## Materials and Methods

### Ethics Statement

The research protocol was approved by the ethical committee for laboratory animal research of the KU Leuven (P029/2008) and was performed according to the Belgian animal welfare regulations and guidelines.

### Animals and Surgical Procedure

**Figure 1:**
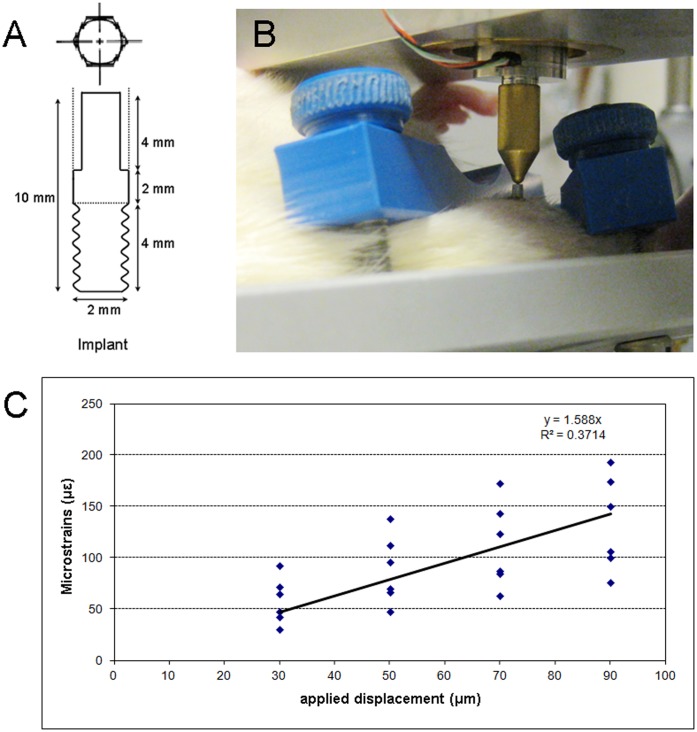
Titanium implants inserted in rat tibiae for *in vivo* loading and *ex vivo* strain gauge measurement. (A) Commercially pure (c.p.) titanium custom-made screw-shaped implant (ISO M2 screw-thread protocol). (B) *In vivo* axial loading applied directly onto the implant. (C) *Ex vivo* strain gauge measurements data on the correlation between the loading magnitude (*i.e*. implant displacement, µm) and the resulting peri-implant strain (µε).

Seventy-five male Wistar rats (3 months old) with an average weight of 349.8 g (S.D. ±7.9) were used in the present study. Out of these, 8 rats were used for defining the desired strain magnitude induced by loading (*ex vivo* load calibration). The remaining 67 rats were used for *in vivo* loading. Custom-made cylindrical implants (ø: 2 mm×L: 10 mm) were obtained from titanium rods (99.6% Ti, Goodfellow Cambridge Ltd., Huntingdon, England). The cylindrical endosseous part of the implant was screw-shaped; the percutaneous part was non-threaded hexagonal (Figure 1A). The implants were cleaned in an ultrasonic bath with distilled water and etched with a solution of HF (4%) and HNO_3_ (20%), resulting in a roughness value (Ra) of 0.45 µm. Implants were sterilized by high-pressure steam heating at 121°C (15 lb./sq. in.) for 20 minutes prior to surgery. The implants were inserted bi-laterally in the medio-proximal site of the tibia.

Implantation was performed under full anesthesia induced by 2.5% isoflurane inhalation (Isoflurane USPR, Halocarbon, NJ, USA). A longitudinal incision was made on the medial side of the proximal tibia and the bone surface was exposed. Perpendicular to the tibial long axis, a cavity was made by perforating both cortices at low rotational speed under constant saline cooling. A surgical drill, 0.3 mm undersized compared to the implant diameter, was used as the final drill for cavity preparation. The implant was inserted manually into the bone using a custom-fit wrench, the wound was closed with resorbable sutures (Vicryl® 3-0, Ethicon, USA). Part of the implant was non-submerged, resulting in percutaneous protrusion and allowing direct access for loading. At the end of the experiment, the animals were sacrificed by cervical displacement under isoflurane-induced anesthesia.

### Ex vivo Strain Gauge Measurement

*Ex vivo* strain gauge measurements were performed to correlate the loading magnitude (*i.e*. implant displacement) with the resulting peri-implant bone strain. For this purpose, 8 rat hind limbs were excised. After exposure of the medial surface of each tibia, an implant was inserted. The limb was placed on a rotating platform and fixated through clamping at the proximal (knee) and distal (ankle) joint. The position of the platform was determined in such a way that the implant and the loading pin were aligned (Figure 1B). A single element strain gauge (type FLG-02-11, TML, Tokyo Sokki Kenkyujo Co., Ltd., Japan) was glued on the exposed bone surface of the tibia, 1 mm above the implant. The lead wires (type 3WP008, Feteris Components BV, UK) were connected at one end to the strain gauge through bondable terminals (TF-2SS, Feteris Components BV, UK) and at the other end to the acquisition system.

Loading was performed by using a custom-made displacement-controlled device [23]. This loading device consisted of a piezo translator (preloaded closed-loop LVPZT translator, P-841.60, ALT, Best, Netherland), which can induce a displacement of up to 120 µm, and a load cell (XFTC 100-M5M-1000N, FGP Sensors, Les Clayes Sous Bois Cedex, France) with a capacity of 1000 N in tension and 100 N in compression. Strain on the surface of peri-implant cortical bone was recorded during displacement of the implants over 30, 50, 70 and 90 µm at a frequency of 1 Hz. The strain reading system included the acquisition of the signal (SCXI 1314, NI, National Instruments, Austin, Texas, USA), amplification, conditioning (SCXI 1520, National Instruments) and transmission to the PC (SCXI 1600 DAQ module, National Instruments). Labview software (Labview 8.6, National Instruments) provided the necessary interface and read-out. The measurements were repeated 5 times with complete removal of the specimen from the device and repositioning. A linear regression analysis was performed to determine the relationship between the applied displacement (µm) and the resulting strain (µε).

### In vivo Mechanical Loading

Rats were randomly allocated to 8 groups, corresponding to 4 loading regimes and 2 experimental periods (Table 1). For each animal, one implant was loaded while the implant in the contralateral limb was unloaded. The loading regimes consisted of high- (40 Hz; HF) and low- (8 Hz; LF) frequency protocols. Within each frequency category, the loading magnitude was defined as such that the maximum induced strain in the high-magnitude loading regime was 2-fold the strain occurring in the low-magnitude protocols. The defined loading frequencies and magnitudes resulted in identical maximum strain rate amplitudes for HF-LM and LF-LM, and for HF-HM and LF-HM. Loading was initiated one day post implant installation, and was applied axially (Figure 1B). The load application took 10 minutes per session and was performed 5 times a week for 1 or 4 weeks, respectively. Anesthesia induced by isoflurane inhalation (Isoflurane USPR, Halocarbon, NJ, USA) was applied during the loading.

**Table 1:** The applied loading regimes, the resulting mean strains and estimated strain rate amplitudes in the peri-implant environment, and the number of animals in each group.

	Loading regime		Mean strain and estimated strain rate amplitude		Group size (n)	
	Frequency (Hz)	Magnitude (µm)	Strain (με)	Strain rate amplitude (με/s)	1-week	4-week
HF-LM	40	8	13	520	9	8
HF-HM	40	16	26	1040	8	8
LF-LM	8	41	65	520	9	9
LF-HM	8	82	130	1040	8	8

HF-LM: high-frequency low-magnitude; HF-HM: high-frequency high-magnitude; LF-LM: low-frequency low-magnitude; LF-HM: low-frequency high-magnitude.

### Specimen Preparation

After sacrifice, the implants and surrounding tissues were isolated and immediately fixed in a CaCO_3_–buffered formalin solution, dehydrated in an ascending series of ethanol concentration and embedded in polymerized methylmethacrylate resin. The tissue blocks containing the implants were sectioned along the longitudinal direction of the tibia and the implant’s axis by a diamond saw (Leica SP1600, Wetzer, Germany). After polishing to a final thickness of 20 to 30 µm (Exakt 400 CS, Exakt Technologies Inc., Germany), the sections were stained with a combination of Stevenel’s blue and Von Gieson‘s picrofuchsin red, visualizing mineralized (red) and non-mineralized (blue) tissues.

### Histology and Histomorphometry

**Figure 2:**
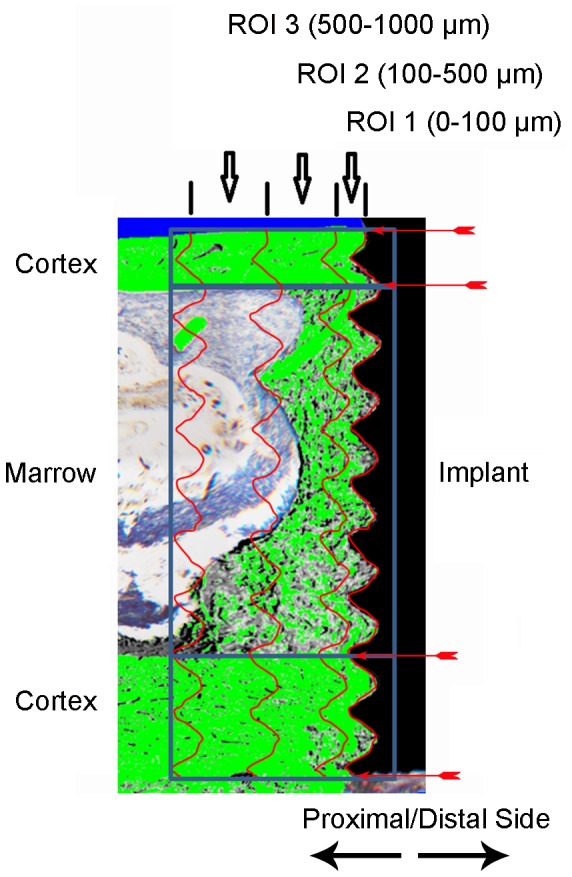
Illustration of the defined regions of interest (ROI) for histomorphometrical analysis of the peri-implant bone. Bone tissue is highlighted in green. The starting and ending points of the cortex and the marrow region that are subject to analysis are indicated by the red arrows. Three ROI’s were defined according to their distance relative to the implant surface: 0–100 µm (ROI1), 100–500 µm (ROI2), and 500–1000 µm (ROI3).

Histological observation and histomorphometrical analyses of the sections were performed under a light microscope (Leica Laborlux, Wetzlar, Germany) equipped with a high sensitivity video camera (AxioCam MRc5, Zeiss, Göttingen, Germany). Histomorphometrical analyses were carried out on both the proximal and distal side of the implant, at the cortical and the medullar level. The following parameters were measured by using an image-analyzing software (Axiovision 4.0, Zeiss, Göttingen, Germany):

- Bone-to-implant contact (BIC, %) = 100 × summation of the lengths of the bone in direct contact with the implant/the implant length from the first till the last bone-to-implant contact.
- Bone fraction (BF, %) = 100 × area occupied by bone/area of the region of interest. Three different regions of interest (ROI) were defined: 0–100 µm (ROI1), 100–500 µm (ROI2) and 500–1000 µm (ROI3) away from the implant surface. The height of all ROI’s was defined by the first till the last bone-to-implant contact (Figure 2).

### Statistical Analysis

Two-way ANOVA followed by Tukey HSD tests was performed to assess the effect of loading and time (*i.e*. the independent variables) on the peri-implant tissue response of BIC (*i.e*. the dependent variable). Three-way ANOVA followed by Tukey HSD tests was performed to assess the effect of loading, time, and region of interest (*i.e*. the independent variables) on the peri-implant tissue response of BF (*i.e*. the dependent variable) (SPSS ver. 13.0, Chicago, IL, USA). Data were reported as mean ± standard error of the mean (SEM). The significance level of *p*<0.05 was acknowledged.

## Results

### Animal and Implant Outcome

Implant surgery and *in vivo* mechanical loading were performed uneventfully for all except 3 implants. A total of 131 samples were obtained, of which 5 were excluded because of peri-implant infection and 3 were lost during histological processing. The remaining 123 samples were successfully processed for histology and histomorphometry.

### Ex vivo Strain Gauge Measurement

The measurements on two limbs were not successful due to technical errors; these were not considered for analysis. For the measurements performed on the remaining 6 limbs, the regression between the applied loading displacement (µm) and the resulting strain (με) was determined (Figure 1C). Based on the established correlation, strains of 13 µε and 26 µε for the HF-LM and HF-HM loading regimes, respectively, were estimated. For the LF-LM and LF-HM loading protocols, strains of 65 µε and 130 µε respectively were induced for the selected loading magnitudes (Table 1).

### Histology

**Figure 3:**
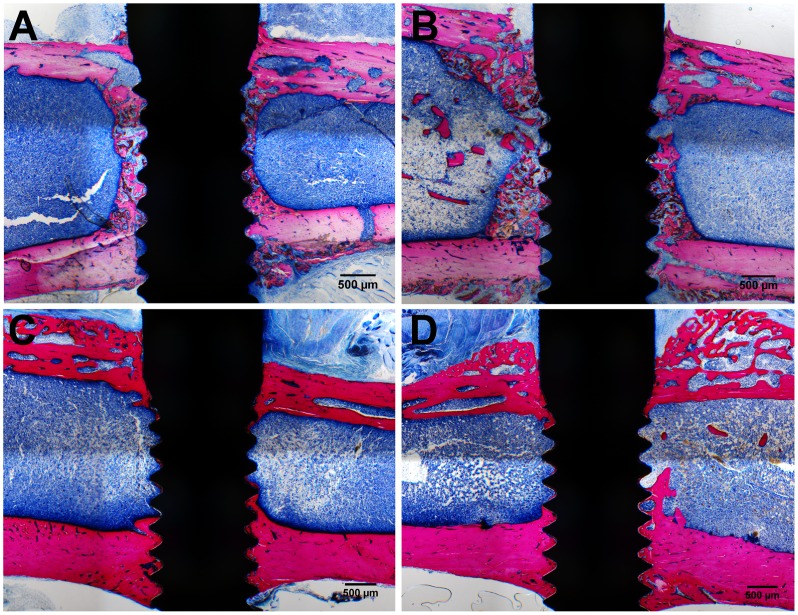
Representative histological sections. Above: for the 1-week experiment, peri-implant bone formation was observed in the medulla around both unloaded (A) and loaded (B) implants. Below: for the 4-week experiment, bone remodeling resulted in a dense bone layer appositioned onto the implant surface in the medullar region for both unloaded (C) and loaded (D) implants.

The histological images revealed bicortical bone apposition to the implant for all the loaded and unloaded implants and for both healing periods (Figure 3). After 1 week, woven bone was formed along the implant surface in the medullar cavity, while remodelling occurred at the peri-implant cortex. After 4 weeks, the newly formed bone in the medulla was remodelled into lamellar bone close to the implant surface. Further, the healing of the peri-implant cortex was complete. No obvious differences between loaded and unloaded implant of the four loading regimes could be noticed on the histological sections.

### Histomorphometry

#### Bone-to-implant contact

**Figure 4:**
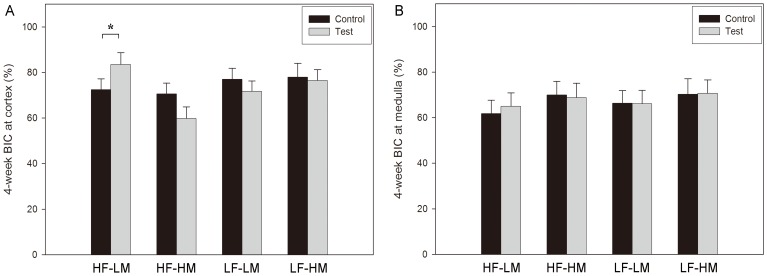
Bone-to-implant contact (BIC) at the cortex (A) and the medulla (B) for the 4-week experiment. Data of the 1-week experiment are not shown as no significant differences were detected (*: *p* = 0.031; ANOVA).

Out of the 4 assessed loading regimes, only cortical BIC was significantly increased in case of HF-LM (40 Hz-8 µm) loading for 4 weeks, compared to the unloaded control (83.49±2.23% *vs*. 72.44±5.47%; loaded *vs.* unloaded; *p* = 0.031, ANOVA) (Figure 4A). No further pronounced loading effect on BIC was detected in the medullar region for the 4 loading regimes (Figure 4B).

Concerning the BIC changes over time, the BIC at the cortical level remained stable (*p*>0.05; ANOVA), whereas a significant increase from 1 to 4 weeks was observed at the medullar level (*p*<0.001; ANOVA). This BIC change was observed in all 4 loading regimes.

#### Bone fraction

The comparison between the unloaded and loaded implant revealed that the peri-implant BF of loaded implants did not significantly differ from the BF of the unloaded implants at both cortical and medullar level and for each loading regime.

As neither loading effect nor interactions between loading and time/ROI were detected for the 4 loading regimes, the BF data of the 4 loading regimes were pooled to assess the overall effect of time (*i.e*. the BF evolution over time) and ROI (*i.e*. the BF distribution in peri-implant region).

**Figure 5:**
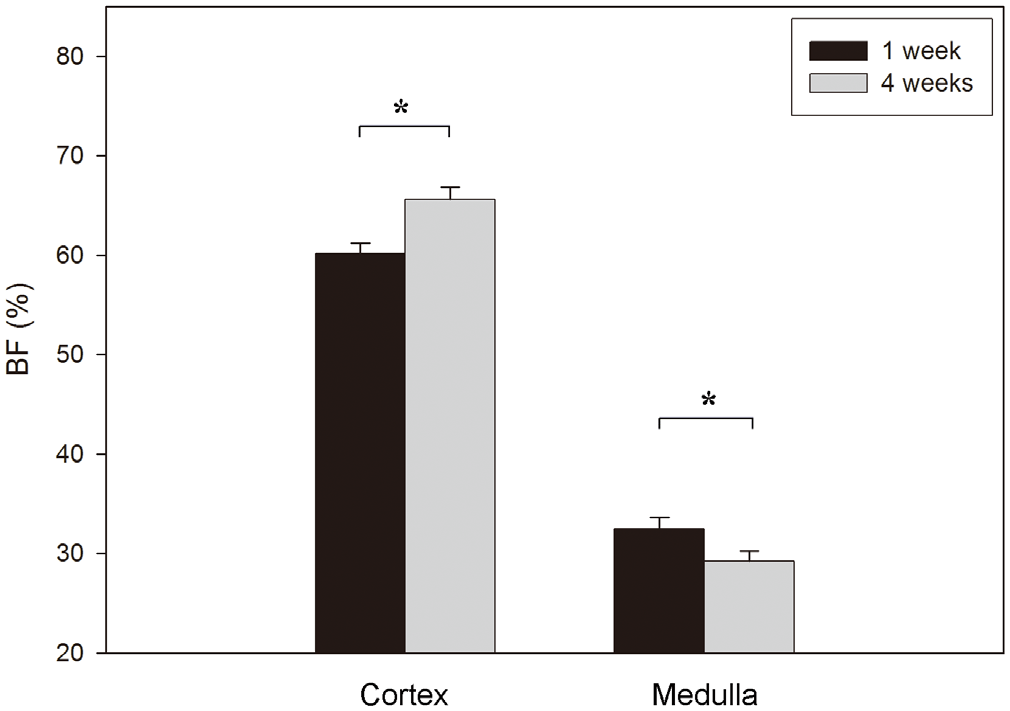
Cortical and medullar bone fraction (BF) evolution from 1 to 4 weeks (*: *p*<0.001; ANOVA).

A significant increase of BF over time was observed at the cortical level (*p*<0.001; ANOVA). Inversely, a significant BF decrease from 1 to 4 weeks was detected at the medullar site (*p*<0.001; ANOVA) (Figure 5).

**Figure 6:**
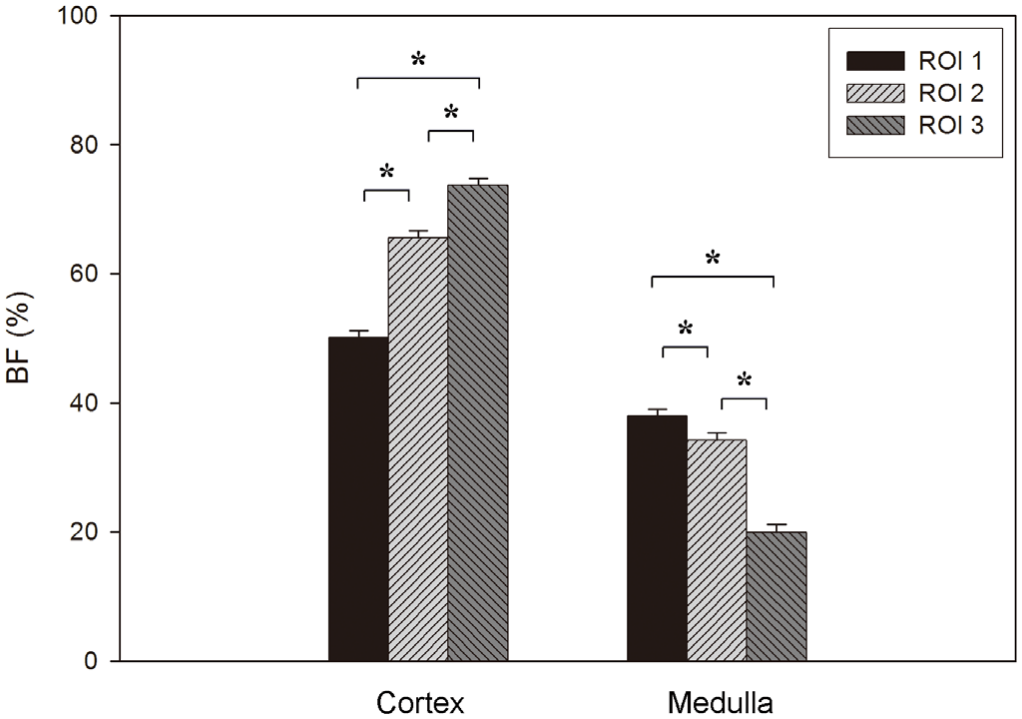
Cortical and medullar bone fraction (BF) distribution in 3 regions of interest (ROI) (*: *p*<0.001; ANOVA followed by Tukey HSD).

With regard to the BF distribution in the peri-implant region, again opposing results were found at cortex and medulla. At the cortex, BF significantly increased at further distance from the implant surface (BF in ROI 1<ROI 2<ROI 3, *p*<0.001; ANOVA followed by Tukey HSD). At the medulla, on the other hand, BF significantly decreased with increasing distance from the implant surface (BF in ROI1 >ROI 2>ROI 3) (*p*<0.001; ANOVA followed by Tukey HSD) (Figure 6).

## Discussion

In the present study, implant osseointegration was assessed under immediate loading at either high- or low-frequency. It was hypothesized that (i) the peri-implant bone responds to high- and low-frequency loading; and that (ii) the response of the peri-implant bone depends on the applied loading magnitude. The main finding of the present study is that bone-to-implant contact is enhanced after 4 weeks of high-frequency low-magnitude loading. This effect, however, was not observed for the respective loading regime after 1 week of loading, or in case of high-frequency high-magnitude loading, or in case of low-frequency loading. Hence, the first study hypothesis is partly confirmed: a response of the peri-implant bone to direct immediate loading was only found in case of HF loading. At the same time, the suggested role of the loading magnitude (2^nd^ hypothesis) on the peri-implant bone response was confirmed.

According to Frost [24], bone (re)modelling is triggered when the tissue deformation (strain) induced by a low-frequency loading exceeds a certain threshold (*i.e*. 1000 µε). On the other hand, when applied at high-frequency, bone can sense and respond to mechanical signals at low magnitudes (*i.e*. from 5 to 10 µε) [2], [25]. Besides the magnitude of the strain, the strain rate amplitude (defined by both loading magnitude and frequency) is considered to be a determining factor for bone response to mechanical loading [26], [27]. In order to define the role of the individual loading parameters (frequency, magnitude – corresponding to the displacement of the loading device, and strain rate amplitude) in immediate implant loading, 4 distinct but comparable loading regimes were defined in this study. The loading parameters were in part chosen based on reports of an anabolic effect of loading on bone [9], [10], [28]. The *ex vivo* calibration data provided information on which implant displacement was required to achieve a certain peri-implant bone strain. Considering the configuration of the loading device, 40 Hz and 8 Hz were selected as high- and low-frequency respectively. For both frequency categories, the loading magnitude was determined in such a way that identical strain rate amplitudes between the two frequency regimes were obtained. In studies from our group [10], [11], [28], the peri-implant strain rate amplitude favoring bone formation was found to be 267 µε/s to 1600 µε/s at low-frequency (3 Hz). In the present study, strain rate amplitudes of 520 µε/s and 1040 µε/s were achieved by loading magnitudes of 41 µm and 82 µm (leading to strains of 65 µε and 130 µε) for a low-frequency regime (8 Hz). Accordingly, for high-frequency loading (40 Hz), the estimated strains of 13 µε and 26 µε induced by loading magnitudes of 8 µm and 16 µm respectively resulted in identical strain rate amplitudes compared with the low-frequency regime. In this way, comparable strain rate amplitudes, anticipated to be anabolic to the peri-implant bone response, were achieved.

Histological observations revealed a normal healing response after implantation, irrespective of the loading regime. These observations were in line with the histomorphometrical data. At the cortex, bone remodelling led to a bone gain in the peri-implant cortex over time (BF increased from 1 to 4 weeks), while the remodelling did not necessarily influence the direct bone contact with the implant (BIC remained stable over time). Compared to the distant host bone, on the other hand, the bone fraction in the direct implant vicinity remained lower, even after 4 weeks of healing. Histologically, the more prominent presence of blood vessels, playing an active role in bone remodelling, in the implant’s vicinity, may explain this lower peri-implant bone fraction. In the peri-implant medullar area – an initially bone-free region – massive woven bone was formed soon after implantation. The formed bone originated from the endosteum of the peri-implant cortex and grew along the implant surface. Subsequent remodelling of this newly formed bone led to less but denser bone, in closer contact with the implant. With regard to the tissue evolution over time in this region, implant osseointegration (quantified as BIC) was found to increase from 1 to 4 weeks, whereas the bone mass (BF) around the implant decreased in the meantime. This is in line with previous findings with the same animal model [15], [16].

The anabolic effects of high-frequency loading have been reported in a number of animal studies [5], [29], [30] and clinical trials [25], [31], [32]. Only few studies, however, investigated the effect of high-frequency loading on bone surrounding implants. De Smet et al. [28], [33] applied high-frequency (30 Hz) loading onto implants 7 days after implantation. They revealed a bone stimulating loading effect in the medullar region. However, no loading effect on implant osseointegration was found. The discrepancy from the findings of the present study (*i.e*. increased cortical osseointegration by HF-LM loading) may owe to the different time of loading. As De Smet *et al*. [28], [33] adopted an implant-healing time of 7 days prior to the loading, the impact of loading on the differentiating cells and tissues in peri-implant region can be diminished, compared to the loading initiated one day after implantation.

In another study, applying a high-frequency loading of 40 Hz directly on the implant, failed to improve the osseointegration [34]. In the mentioned study, however, a moment was applied instead of an axial force. Taking into account the detrimental effect of excessive micromotion and shear strains on osseointegration [7], it is logical that, in case of screw-shaped implants, load transfer from the implant to the surrounding tissues is more favorable in case of axial compared to rotational loading.

In the current experiment, HF-LM immediate loading was found to enhance bone-to-implant contact at the peri-implant cortex after loading for 4 weeks. Meanwhile, the high-magnitude loading at the same high-frequency failed to do so. In this respect, low-magnitude loading was better to promote cortical osseointegration. Furthermore, the gain in peri-implant cortical bone mass over time can be attributed to the inherent healing of the host tissue; neither low- nor high-magnitude loading was found to contribute significantly. Similar insignificant findings were observed in the peri-implant medulla. Potential loading effects are likely to be overruled by this active tissue repair and remodeling.

Similar to high-frequency loading, the effect of low-frequency loading on bone adaptation and regeneration has also been acknowledged [2], [5]. Well-controlled mechanical loading at low-frequency, when applied directly to the implant, either in an early or immediate loading protocol, can improve bone formation in the peri-implant region and implant osseointegration [7], [8], [9], [12], [13]. Relatively small loading displacements were selected for the low-frequency loading regime in the current study. The resultant deformations by the loading were far below the (re)modelling threshold of 1000 µε recommended by Frost [24]. Although the applied strain rate amplitudes were identical to the ones in high-frequency loadings and considered osteogenic [11], [28], no significant effect of low-frequency loading on implant healing was found in the present experiment. The implication might be that (1) to induce a positive bone response to mechanical loading, at least one of the constituting elements of loading (*i.e*. magnitude or frequency) needs to go beyond a certain threshold; (2) after fulfillment of the above condition, the impact of loading element combination (*i.e*. strain rate amplitude) on bone response can be considered.

The exact mechanism of how mechanical loading affects bone is yet unclear. Compared to the high-frequency loading, the host tissue perceiving the low-frequency loading is more dependent on the loading magnitude. When keeping loading frequency and the number of loading events constant, variations in strain magnitude can explain differences in the osteogenic response to the low-frequency loading. *I.e*. the larger the deformations generated in the bone, the greater the increases in bone mass [18], [19], [20], [21]. This can be interpreted with relatively simple models such as the mechanostat [24] and the fluid flow theory [35]. According to the fluid flow theory, the load-induced fluid shear stress acts as the signal activating bone remodelling. The signal acts on osteocytes and cell processes in the lacunar-canalicular system. Therefore, the anabolic effect of low-frequency loading on bone is dependent on the loading magnitude which in turn affects the load-induced strain.

Under the high-frequency regime, the loading can also be sensed by the bone [22], [36]. Local strains on the tibia surface have been recorded to be less than 10 µε in case of whole body vibration [5], [37]. Apparently, the induced strain is extremely small, far less than the (re)modelling threshold of 1000 µε for the low-frequency loading [24]. Therefore, the notion has been called up that the anabolic response of the host tissue to the high-frequency loading was mainly dependent on the loading frequency, rather than the loading magnitude [5], [36], [38]. Explanations of this dependence were suggested by the theoretical models of You et al. [39] and Han et al. [40]. Their models were based on the facts that (1) osteocyte processes were attached along their length by tethering filaments, and (2) the actin filament bundle in dendritic processes led to a highly polarized cell whose processes were several hundred times stiffer than the osteocyte cell body [40]. Hence, the flow-induced drag on these filaments would produce a tension that could greatly amplify the very small whole tissue strains at the cellular level. By predicting the strain amplification ratio from the tissue to the cell level, they found that this amplification ratio not only increased with loading frequency, but also decreased with loading magnitude [39]. Therefore, under high-frequency loading, low bone strains were amplified most, suggesting a more efficient mechanotransduction due to the cellular perception of the high-frequency signals. This “less is more” phenomenon is also supported by the findings of the high-frequency loadings in the present study.

The frequency referred to as low frequency in this study is in fact higher than 1 to 3 Hz, which is more commonly used as low frequency in other studies. Accordingly, the high strain (130 µε) considered in this study is lower compared to what is usually referred to as high strain [9], [10], [11], [17], [41]. Nevertheless, as the loading parameters are interpreted relative to each other in this study, 8 Hz is considered to be the low frequency and 130 µε is considered as the high strain. Meanwhile, the amount of loading cycles of high- and low-frequency regimes was differing by 5 fold (*i.e*. 24000 *vs*. 4800 cycles for the loading duration of 10 minutes). Considering the above, further research of loading with lower frequency (*e.g*. 1 Hz) and constant loading cycles would be valuable to comprehend the role of high- *vs*. low-frequency loading on peri-implant bone. Another limitation to this study was that the exact mechanical environment at the implant interface remains unknown. More numerical modelling research is needed to identify the detailed mechanics of the bone-to-implant interface.

Although implant therapy is a successful treatment [42], there is room for improvement in case of compromised bone conditions (*e.g*. diabetic, osteoporotic, or irradiated bone). Acceleration of the osseointegration process to allow earlier function of the implant would also be welcomed by the clinical community. Whole body vibration proved its highly significant osteogenic potential around implants [14], [15], [16], but has the disadvantage that the ruling mechanical conditions in terms of load magnitude and frequency are poorly controlled. This study aimed to overcome this flaw by controlling load magnitude and frequency, but failed to induce such an explicit histological response to the mechanical stimulation compared to whole body vibration. The exact bone-stimulating mechanical environment as in case of whole body vibration could therefore not be mimicked by this direct loading protocol. The closest we get in understanding the local mechanical conditions, is by means of extrapolation of *ex vivo* strain gauge data towards the interface [43] and through numerical modelling [44], [45].

In conclusion, the stimulatory effect of immediate implant loading on bone-to-implant contact was only observed in case of high-frequency (40 Hz) low-magnitude (8 µm) loading. The applied load regimes failed to influence the peri-implant bone mass.
